# Does campus sports environment matter for physical fitness? The mediating roles of peer support and perceived physical literacy

**DOI:** 10.3389/fpsyg.2026.1822645

**Published:** 2026-04-20

**Authors:** Mingliang Song, Qishun Yang

**Affiliations:** 1 School of Football, Wuhan Sports University, Wuhan, China; 2 School of Physical Education, Wuhan Business University, Wuhan, China

**Keywords:** chain mediation, peer support, perceived campus sports environment, perceived physical literacy, physical fitness

## Abstract

**Purpose:**

Physical inactivity and declining physical fitness among Chinese college students remain important public health concerns. This study examined whether perceived campus sports environment was prospectively associated with physical fitness through peer support and perceived physical literacy, and explored gender differences in key variables.

**Methods:**

A two-wave prospective observational study was conducted among 1,165 undergraduates (56.3% female; M_age = 19.1 ± 0.89) from five universities in Hubei Province, China. At T1, participants completed measures of perceived campus sports environment, peer support, and perceived physical literacy. At T2, physical fitness was assessed according to the National Student Physical Fitness Standards using sex-specific standardized item scores and the official composite scoring procedure. Descriptive statistics, Pearson correlations, independent-samples t tests, and serial mediation analysis using PROCESS Model 6 with 5,000 bootstrap samples were conducted.

**Results:**

Male students reported significantly higher perceived facility access and peer-support indicators than female students, whereas no significant gender differences were observed in perceived physical literacy or physical fitness. Perceived campus sports environment (*r* = 0.316), peer support (*r* = 0.475), and perceived physical literacy (*r* = 0.662) were all positively associated with physical fitness. Mediation analysis indicated that the association between perceived campus sports environment and physical fitness was indirectly linked through peer support and perceived physical literacy. The indirect pathway via perceived physical literacy accounted for the largest proportion of the total effect (42.42%), followed by the serial pathway through peer support and perceived physical literacy (25.60%) and the pathway via peer support alone (17.58%).

**Conclusion:**

These findings suggest that campus sports environment, peer support, and perceived physical literacy should be considered together when interpreting differences in university students’ physical fitness. Strategies that foster supportive campus sport cultures and strengthen students’ perceived physical literacy may help support physical fitness in university settings, with particular attention to female students’ relatively lower perceived facility access and peer-support indicators.

## Introduction

In recent years, insufficient physical activity among adolescents has emerged as a critical public health issue worldwide. According to the World Health Organization’s 2020 report, over 80% of adolescents globally fail to meet the recommended levels of physical activity (Guthold et al., 2020). Similar patterns have been reported among college students (Pengpid et al., 2015), including those in Asian populations (Suminski et al., 2002; Seo et al., 2012). In China, data from the Ministry of Education’s Eighth National Student Physical Fitness Survey indicate a consistent decline in college students’ physical fitness levels (Department of Physical Health And Arts Education, Ministry of Education, 2021). Specifically, endurance performance, as measured by the 1,000-meter run for males and the 800-meter run for females, has declined in recent years (Hou, 2017), and muscular strength remains weak (He and Shi, 2019). These trends have led to a rising prevalence of age-related chronic conditions, such as obesity, hypertension, and diabetes, among college students (Hou et al., 2021; Shao et al., 2019). This deterioration not only poses significant threats to individual health, but also exacerbates the burden of chronic disease and associated healthcare costs at the societal level (Bianco et al., 2018).

Evidence suggests that physical activity habits and fitness levels established during adolescence have long-term implications for lifelong health and exercise engagement (Hulteen et al., 2017). As such, improving adolescent physical fitness has become a core agenda item in the “Healthy China 2030” national strategy (Dong et al., 2020; Mao et al., 2018). In response to this challenge, the Chinese government and educational authorities have introduced several policies, including the Healthy China 2030 Plan Outline, the Guidelines for Health Education in Higher Education Institutions, and the Opinions on Strengthening and Improving School Physical Education in the New Era. These policies aim to improve the campus physical activity environment through three major initiatives: (1) mandating 1 h of daily on-campus exercise (Liang et al., 2018); (2) incorporating physical education outcomes into the evaluation framework for “Double First-Class” universities (Zhong and Li, 2022); and (3) systematically reforming physical education curricula, campus sports competitions, and fitness assessment systems (State Council Office of the People’s Republic of China, 2016; Xiang et al., 2023).

While these structural policies have brought about significant changes in the campus sports environment, studies show that simply creating supportive environments does not necessarily lead to increased physical activity. Only when students develop and internalize subjective perceptions of these environments can they enhance self-efficacy or build behavioral habits that translate policy into meaningful action (Rhodes et al., 2020).

From a socio-ecological perspective, college students’ exercise behaviors are shaped by interactions across multiple levels, including individual factors such as exercise cognition and perceived physical literacy, interpersonal influences such as peer and teacher interactions, institutional conditions such as campus sport policies and facilities, and broader cultural and policy contexts (Bronfenbrenner, 2005; Stokols, 1996). Within this framework, perceived campus sports environment, defined as students’ subjective evaluations of accessibility, institutional support, and cultural inclusiveness, may be understood as a proximal student-level indicator of how the campus sport context shaped by institutional resources and policies is experienced (Gong, 2021; Reid et al., 2015). This construct captures how students interpret and experience objective environmental resources and may therefore be associated with subsequent participation in physical activity and, indirectly, with physical fitness outcomes.

University-level policies aimed at enhancing curricula, strengthening faculty, improving facilities, and refining institutional mechanisms have created a favorable external environment for student engagement in sports (Gong, 2021). However, it is students’ perceived sports environment that reflects the effectiveness of these policies at the individual level. Students who report more favorable perceptions of their campus physical activity environment may be more likely to report stronger exercise motivation (Liu, 2007), higher perceived physical literacy (Demetriou et al., 2018), and higher physical fitness at follow-up. In this regard, perceived campus sports environment may reflect the likelihood of student participation in physical activity and may also be associated with later physical fitness outcomes (Gong et al., 2021).

Most existing studies have focused on the direct effects of school physical activity environments on students’ participation in physical activity (Gong et al., 2021; Gou, 2022). However, few studies have explored how these environments may be indirectly associated with students’ physical fitness through psychosocial mechanisms (e.g., peer support) and capacity-building processes (e.g., physical literacy). According to socio-ecological systems theory, individual behaviors are embedded within multilayered environmental contexts (Bronfenbrenner, 1979). Peer support, as a crucial component of the social environment, plays a unique role in shaping college students’ engagement in physical activity. Research indicates that exercising with peers and receiving emotional encouragement can significantly enhance self-efficacy for physical activity (Douglas et al., 2024; Wang et al., 2018), while a lack of peer support is associated with a higher risk of dropping out of exercise routines (Zhang Y. et al., 2022). Moreover, peer interactions may contribute to the development of physical literacy (Chen et al., 2024), which encompasses the knowledge, skills, motivation, and confidence necessary for lifelong physical activity engagement (Capel and Whitehead, 2012). Empirical studies have shown a significant positive correlation between peer support and physical literacy, with students receiving greater peer support reporting higher levels of physical literacy (Chen et al., 2024; Douglas et al., 2024). In addition, peer support not only serves as a motivational source for maintaining physical activity habits but also functions as a long-term social support network for the development and consolidation of physical literacy (Gruber, 2008).

Students with higher levels of perceived physical literacy may be more likely to understand the value of physical activity, feel confident in exercise-related settings, and report greater readiness to engage in regular activity. Physical literacy has been found to be closely associated with both physical fitness and physical activity behaviors among college students (Long et al., 2024). Those with higher levels of physical literacy tend to perform better in physical fitness assessments, and indicators such as physical capacity, activity frequency, and exercise duration are positively correlated with physical literacy scores (Luo et al., 2022). Moreover, physical literacy has been shown to significantly predict multiple fitness outcomes, including aerobic endurance, muscular strength, and flexibility (Long et al., 2024). By influencing moderate-to-vigorous physical activity (MVPA), physical literacy may support both the initiation and maintenance of physical activity, which may in turn be associated with better physical fitness outcomes (Pastor-Cisneros et al., 2021).

However, existing research presents three main limitations. First, most studies examine isolated variables (e.g., physical environment or physical literacy) without systematically testing multilevel mediation mechanisms. Second, measurement approaches to physical literacy remain heterogeneous. Some studies emphasize physical competence or motor performance, whereas others rely on brief self-report or perceived physical literacy instruments that do not capture the full multidimensional construct. Third, peer-related processes may operate differently in Chinese university settings, yet this possibility remains insufficiently examined in context-specific research.

**Figure 1:**
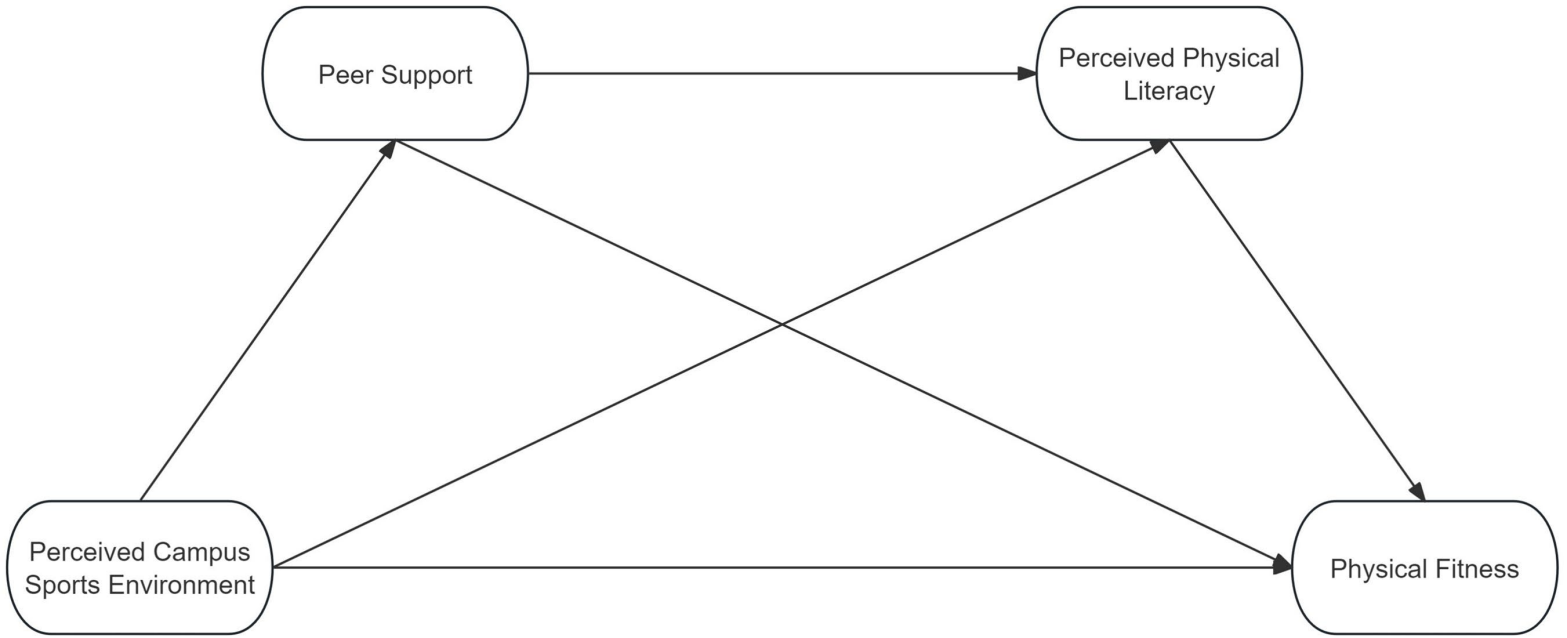
Hypothesized model.

Based on the above gaps, the present study aims to examine a chained mediation mechanism linking perceived campus sports environment, peer support, perceived physical literacy, and physical fitness among college students (see Figure 1). Specifically, the study investigated whether perceived campus sports environment was prospectively associated with physical fitness through peer support and perceived physical literacy. This research contributes to a deeper understanding of how students’ perceptions of the campus physical environment may be associated with physical fitness through psychosocial and capacity-building pathways.

## Materials and methods

### Participants and procedure

This study used a two-wave prospective observational design and was conducted among undergraduate students from five universities in Hubei Province, China, between May and November 2024. Participants were included if they were non-physical education majors aged 18 to 22 years and were excluded if they were sports-specialty students or enrolled in physical education programs.

At baseline (T1; May–October 2024), participants completed an online questionnaire assessing demographic characteristics, perceived campus sports environment, peer support, and perceived physical literacy. At follow-up (T2; November 2024), physical fitness data were obtained from the university’s routine annual physical fitness assessment, administered in accordance with the National Student Physical Fitness Standards.

**Figure 2:**
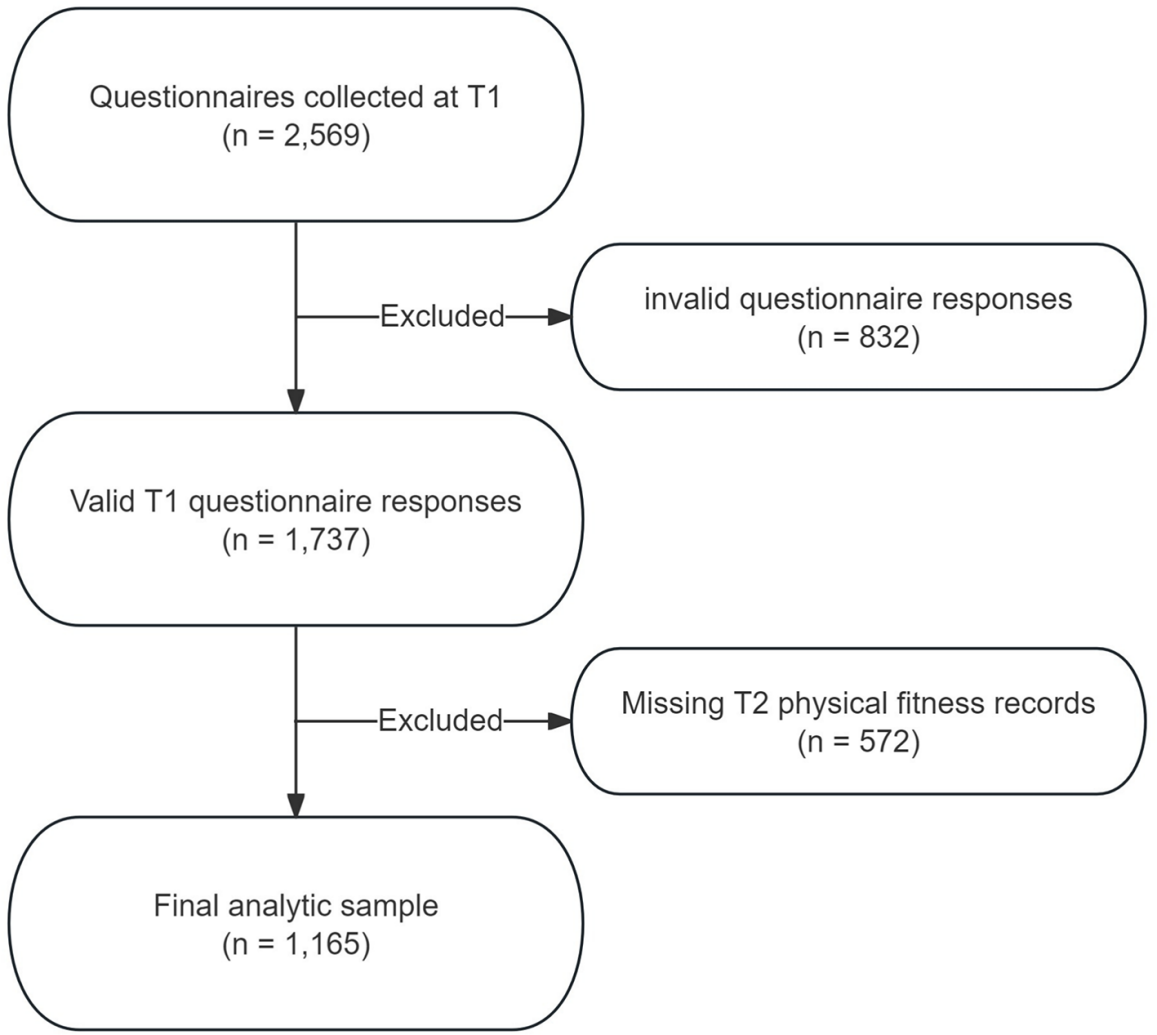
Flow diagram of sample selection and two-wave follow-up procedure.

A total of 2,569 questionnaires were collected at T1. After excluding 832 invalid questionnaire responses, 1,737 participants remained eligible for follow-up matching. At T2, physical fitness records were unavailable for 572 participants. Consequently, 1,165 participants with valid T1 questionnaire data and matched T2 physical fitness records were retained in the final analytic sample (see Figure 2).

This study was reviewed and approved by the Academic Committee of the School of Physical Education, Wuhan Business University. The study was conducted in accordance with the Declaration of Helsinki. All participants were informed of the purpose of the study, the voluntary nature of participation, and the anonymous handling of the data before participation. Informed consent was obtained prior to participation, and questionnaire responses and physical fitness records were anonymized before analysis.

### Instruments

#### Perceived campus physical activity environment

Perceived campus physical activity environment was assessed using the Physical Education Environment Perception Scale, which was revised by Gong Qingbo on the basis of Zhao Xiaoyang’s College Student Development Survey Scale (Zhao, 2014). The scale originally consisted of 19 items across four dimensions: curriculum structure (e.g., “The amount and intensity of practice required by the physical education program is reasonable for you”), teacher guidance (e.g., “The teacher adopts inspirational and interactive teaching methods in the physical education classroom”), institutional support (e.g., “The physical education guidance and training provided by your institution is adequate”), and facility access (e.g., “The school provides good exercise facilities”). Two items were removed due to low factor loadings, resulting in a final 17-item instrument. Items are rated on a 5-point Likert scale ranging from 1 (strongly disagree) to 5 (strongly agree). Previous studies have confirmed that this scale exhibits good structural validity and reliability (Gong et al., 2021; Wu et al., 2024). In the current study, the Cronbach’s *α* values for the four dimensions were 0.926, 0.938, 0.885, and 0.893, respectively, with an overall reliability coefficient of 0.912. The Kaiser–Meyer–Olkin (KMO) value was 0.913, and item factor loadings ranged from 0.806 to 0.885.

#### Peer support

Peer support was measured using the scale adapted by Verloigne et al. (2016) from Deforche et al. (2010). The original instrument operationalized peer-related social influences as three separate indicators: peer modeling, peer co-participation, and peer encouragement, rather than as a conventional multi-item latent scale. In the present study, only these peer-related items were used, with the items asking how often friends were physically active, physically active together with the participant, and encouraging the participant to be physically active. Each item was rated on a 5-point Likert scale ranging from 1 (never) to 5 (always). This type of brief peer-support measure has also been applied in recent Chinese adolescent research on peer support and exercise-related behavior (Zou et al., 2023). Because the present measure consisted of only three closely related but conceptually distinct activity-related indicators, we treated it as a brief composite representation of activity-related peer support rather than as a full reflective latent construct. Accordingly, exploratory factor analysis and internal consistency were used as pragmatic checks of whether the three indicators could reasonably be summarized in the present sample. All three items loaded strongly on a single factor (0.875–0.912), and internal consistency was acceptable (Cronbach’s *α* = 0.867). Nevertheless, this brief measure should be interpreted as capturing limited activity-related peer support rather than the full multidimensional construct of peer support.

#### Perceived physical literacy

Perceived physical literacy was assessed using the Perceived Physical Literacy Instrument (PPLI) developed by Sum et al. (2018). The scale comprises nine items across three dimensions, consistent with the original validation study: knowledge and understanding (e.g., “I am aware of the health benefits of sports”), self-expression and communication with others (e.g., “I have strong social skills”), and sense of self and self-confidence (e.g., “I possess self-management skills for fitness”). Items were rated on a 5-point Likert scale ranging from 1 (strongly disagree) to 5 (strongly agree). The Cronbach’s *α* values for knowledge and understanding, self-expression and communication with others, and sense of self and self-confidence were 0.859, 0.867, and 0.891, respectively, with an overall Cronbach’s *α* of 0.894. The KMO value was 0.855, and standardized factor loadings ranged from 0.809 to 0.893.

For brevity, these three dimensions are hereafter referred to as knowledge and understanding, self-expression/communication, and self-confidence, respectively. In this study, the PPLI was used to assess perceived physical literacy rather than the full multidimensional construct of physical literacy.

#### Physical fitness

Physical fitness was evaluated based on the National Student Physical Fitness Standards (University Level) (Dong et al., 2023). In accordance with the university’s education plan, all undergraduates undergo an annual physical fitness assessment. The indicators included body mass index (BMI), vital capacity, 50-m sprint, sit-and-reach, standing long jump, pull-ups (men)/1-min sit-ups (women), and 1,000-m run (men)/800-m run (women) (Zhang C. et al., 2022). According to the national standard, raw test results were converted into sex-specific standardized item scores, which were then combined using the official weighting scheme to generate a standardized total physical fitness score out of 100. Participants’ scores were classified according to national criteria: ≥90.0 as “Excellent,” 80.0–89.9 as “Good,” 60.0–79.9 as “Pass,” and <60.0 as “Fail” (Yang et al., 2024).

### Statistical analyses

Data were analyzed using SPSS 27.0 and AMOS 28.0. Confirmatory factor analyses (CFA) were conducted for perceived campus sports environment and perceived physical literacy, both of which demonstrated acceptable model fit (see Table 1). Because peer support was assessed using three items and treated as an observed composite rather than a latent construct, exploratory factor analysis and internal consistency were used to evaluate its dimensionality and reliability. Skewness and kurtosis values fell within acceptable thresholds (±2 and ±7, respectively) for all variables, indicating that the data were approximately normally distributed (Kline, 2016).

**Table 1:** Confirmatory factor analysis results for each scale.

Scale	CMIN/DF	RMSEA	SRMR	IFI	TLI	CFI	GFI
Perceived campus sports environment	2.688	0.038	0.0184	0.987	0.984	0.987	0.969
Perceived physical literacy	3.339	0.045	0.0197	0.992	0.987	0.992	0.976

Descriptive statistics were computed for all study variables. Independent-samples t tests were used to examine gender differences, and Cohen’s d was reported as an index of effect size. Pearson’s correlation coefficients were used to assess the bivariate associations among key variables. Finally, a serial mediation model was tested using PROCESS macro v4.2 for SPSS (model 6, Hayes, 2013), with perceived campus sports environment as the predictor, peer support and perceived physical literacy as sequential mediators, and physical fitness at follow-up as the outcome. Age and gender were included as covariates. Bias-corrected bootstrap confidence intervals were based on 5,000 resamples. Statistical significance was set at *p* < 0.05. Following Cohen (1988), correlation coefficients of 0.10–0.29 were interpreted as small, 0.30–0.49 as medium, and 0.50 or higher as large.

## Results

### Common method bias test

Because perceived campus sports environment, peer support, and perceived physical literacy were all assessed using self-report measures at T1, common method bias could not be ruled out completely. Harman’s single-factor test was performed as a preliminary diagnostic. The exploratory factor analysis identified eight factors with eigenvalues greater than 1. Moreover, the first factor accounted for 13.996% of the total variance, which was below the commonly used 40% threshold (Tang and Wen, 2020). These findings reduce the likelihood that a single common factor accounted for the observed covariance among the self-reported variables. However, Harman’s test is only a preliminary diagnostic rather than a definitive test of method bias. Therefore, common method bias cannot be ruled out completely, and the findings should still be interpreted cautiously.

### Descriptive statistics and differences

A total of 1,165 undergraduate students participated in the study (56.3% female; M_age = 19.1 ± 0.89). Male students reported significantly higher scores than female students on perceived facility access (*t* = 2.222, Cohen’s *d* = 0.131), peer modeling (*t* = 2.716, Cohen’s *d* = 0.16), peer co-participation (*t* = 2.411, Cohen’s *d* = 0.142), and peer encouragement (*t* = 3.209, Cohen’s *d* = 0.19) (see Table 2).

**Table 2:** Descriptive statistics and gender comparisons.

Domain	Subdimension	Male (*n* = 509)	Female (*n* = 656)	*t*	Cohen’s *d*
Perceived campus sports environment	Curriculum structure	3.09 ± 0.76	3.07 ± 0.79	0.339	0.02
	Teacher guidance	3.13 ± 0.78	3.18 ± 0.75	−1.083	−0.064
	Institutional support	3.11 ± 0.78	3.1 ± 0.76	0.149	0.009
	Facility access	3.04 ± 0.83	2.93 ± 0.77	2.222*	0.131
Peer support	Peer modeling	3.18 ± 0.78	3.05 ± 0.84	2.716*	0.16
	Peer co-participation	3.16 ± 0.73	3.05 ± 0.8	2.411*	0.142
	Peer encouragement	3.02 ± 0.79	2.86 ± 0.87	3.209*	0.19
Perceived physical literacy	Knowledge and understanding	2.98 ± 0.8	2.89 ± 0.8	1.848	0.109
	Self-expression/communication	3.05 ± 0.8	2.99 ± 0.8	1.174	0.069
	Self-confidence	3.02 ± 0.72	3.01 ± 0.77	0.281	0.017
Physical fitness		72.72 ± 10.61	72.06 ± 11.08	1.032	0.061

Values are presented as mean ± SD. *p < 0.05. Cohen’s *d* indicates effect size.

### Correlation analysis

**Figure 3:**
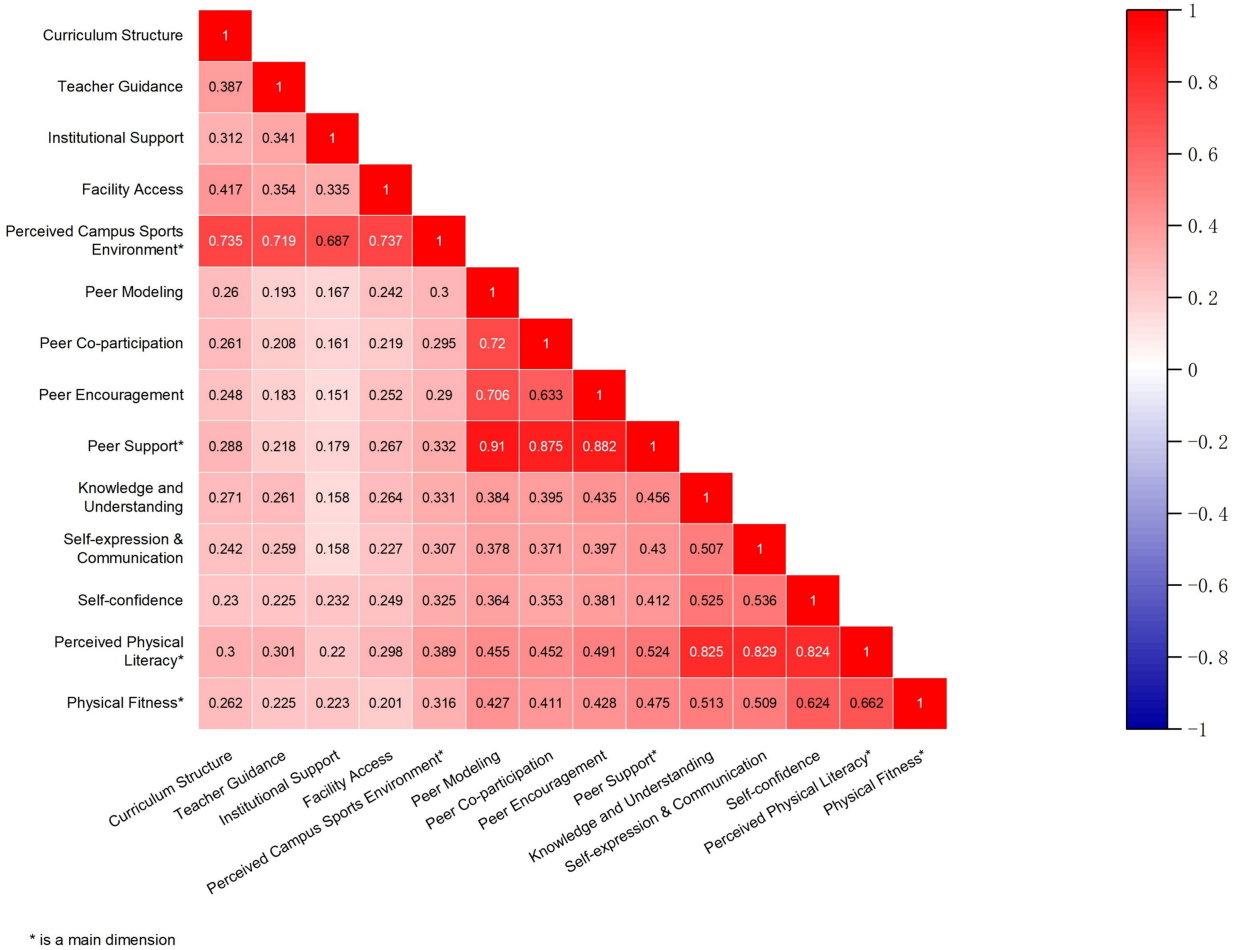
Correlation heatmap among main study variables.

As shown in Figure 3, Pearson’s correlation analysis revealed that all dimensions of perceived campus sports environment (curriculum structure: *r* = 0.262; teacher guidance: *r* = 0.225; institutional support: *r* = 0.223; facility access: *r* = 0.201; overall: *r* = 0.316), peer support (peer modeling: *r* = 0.427; peer co-participation: *r* = 0.411; peer encouragement: *r* = 0.428; overall: *r* = 0.475), and perceived physical literacy (knowledge and understanding: *r* = 0.513; self-expression/communication: *r* = 0.509; self-confidence: *r* = 0.624; overall: *r* = 0.662) were significantly and positively associated with physical fitness. These results indicate that perceived campus sports environment, peer support, and perceived physical literacy were each positively associated with college students’ physical fitness.

### Chain mediation analysis

Table 3 presents the results of the path regression results from the chain mediation model. Model 1 showed that perceived campus sports environment was significantly and positively associated with physical fitness (*β* = 0.312, *p* < 0.001). In Model 2, perceived campus sports environment was significantly and positively associated with peer support (*β* = 0.323, *p* < 0.001), while gender showed a small but significant negative coefficient (*β* = −0.082, *p* < 0.01). Model 3 revealed that both perceived campus sports environment (*β* = 0.237, *p* < 0.001) and peer support (*β* = 0.442, *p* < 0.001) were significantly and positively associated with perceived physical literacy. In Model 4, peer support (*β* = 0.17, *p* < 0.001) and perceived physical literacy (*β* = 0.558, *p* < 0.001) were both significantly and positively associated with physical fitness. The direct association between perceived campus sports environment and physical fitness was no longer statistically significant (*β* = 0.045, *p* > 0.05), which was consistent with the possibility of an indirect association through peer support and perceived physical literacy. Collinearity diagnostics in the final model suggested no problematic multicollinearity, with tolerance values ranging from 0.671 to 0.821 and VIF values ranging from 1.219 to 1.490.

**Table 3:** Regression results for the chain mediation model.

Model	Outcome variable	Predictor variable	Fit index			Regression coefficients		
			*R*	*R* ^2^	*F*	*β*	*t*	95% CI for B
1	Physical fitness	Perceived campus sports environment	0.318	0.101	43.566	0.312	11.119***	(4.993, 7.132)
		Gender				−0.023	−0.814	(−1.697, 0.702)
		Age				0.029	1.027	(−0.325, 1.038)
2	Peer support	Perceived campus sports Environment	0.347	0.12	52.879	0.323	11.659***	(0.346, 0.486)
		Gender				−0.082	−2.974**	(−0.198, −0.040)
		Age				0.054	1.936	(−0.001, 0.089)
3	Perceived physical literacy	Perceived campus sports environment	0.573	0.329	141.978	0.237	9.247***	(0.215, 0.331)
		Peer support				0.442	17.234***	(0.351, 0.441)
		Gender				0.008	0.342	(−0.051, 0.073)
		Age				0.044	1.794	(−0.003, 0.067)
4	Physical fitness	Perceived campus sports environment	0.68	0.463	199.789	0.045	1.888	(−0.034, 1.779)
		Peer support				0.17	6.59***	(1.799, 3.324)
		Perceived physical literacy				0.558	21.25***	(8.542, 10.28)
		Gender				0.007	0.316	(−0.782, 1.082)
		Age				−0.018	−0.822	(−0.750, 0.307)

**p < 0.01, ***p < 0.001. *β*, standardized regression coefficient; *B*, unstandardized regression coefficient.

**Figure 4:**
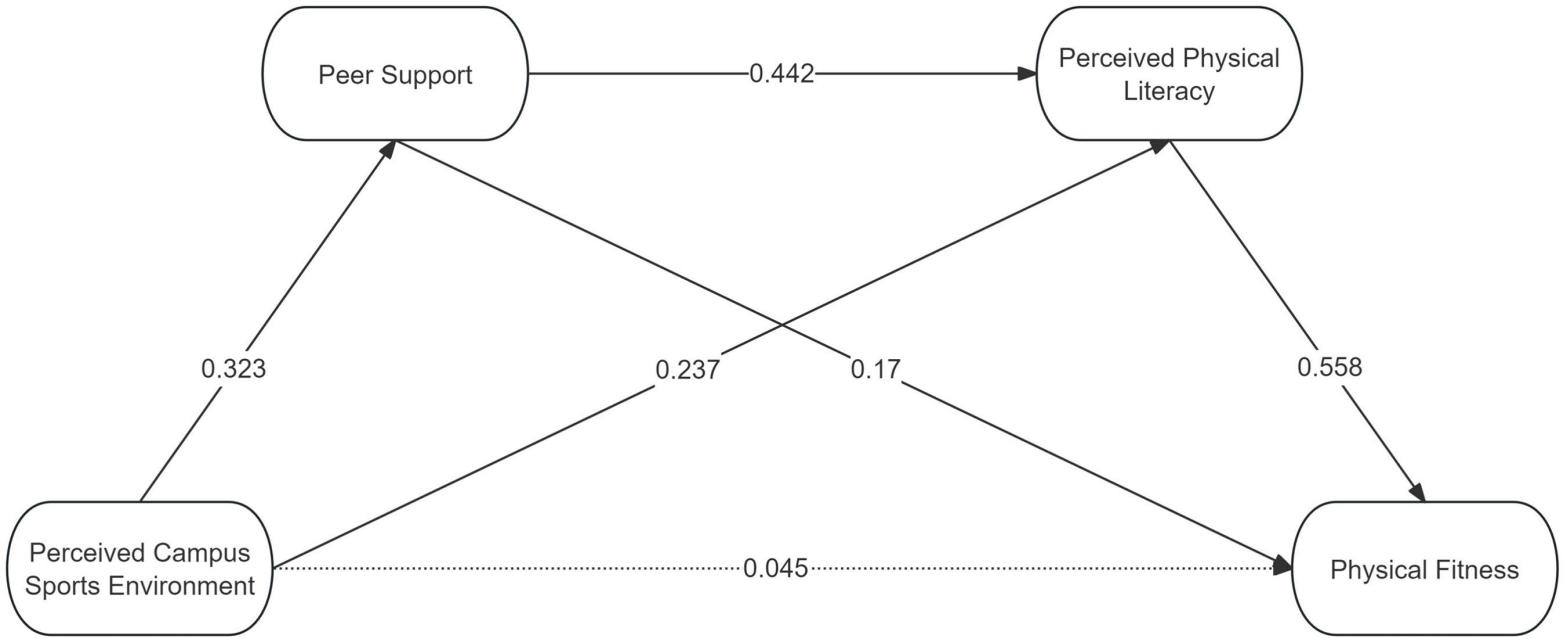
Estimated chain mediation model.

Figure 4 further visualizes this chain mediation model, illustrating the indirect pathways through peer support and perceived physical literacy.

To further examine the hypothesized indirect associations, a bias-corrected percentile bootstrap method was applied. As shown in Table 4, all indirect paths were statistically significant because their 95% confidence intervals did not include zero. The simple mediation of perceived physical literacy accounted for the largest proportion of the total effect (42.42%), followed by the chain mediation of peer support and perceived physical literacy (25.60%), and the simple mediation of peer support (17.58%). Overall, the indirect effects accounted for 85.62% of the total effect, supporting the plausibility of the hypothesized chained pathway.

**Table 4:** Bootstrap estimates of indirect effects and proportions.

Effect type	Effect	SE	LLCI	ULCI	Proportion of total effect
Total effect	6.063	0.545	4.993	7.132	
Direct effect	0.872	0.462	−0.034	1.779	
Total indirect	5.191	0.438	4.352	6.08	85.62%
Indirect 1 (via peer support)	1.066	0.2	0.691	1.474	17.58%
Indirect 2 (via perceived physical literacy)	2.572	0.299	1.992	3.172	42.42%
Indirect 3 (via peer support → literacy)	1.552	0.191	1.205	1.952	25.60%

LLCI, lower limit of the 95% confidence interval; ULCI, upper limit of the 95% confidence interval. An indirect effect was considered statistically significant when the 95% confidence interval did not include zero.

## Discussion

### Gender differences

This study found that male students reported significantly higher scores in perceived facility access and several peer-support indicators than female students, a pattern that is broadly consistent with previous research (Sheng et al., 2025; Zhou, 2024). Several sociocultural and environmental factors may partly explain these gender differences. Prior studies suggest that gender norms can shape sport preferences, participation opportunities, and exercise-related socialization. This may partly account for the pattern observed in the present study (Brazo-Sayavera et al., 2021). Some studies also suggest that perceived accessibility, comfort, and safety of recreational spaces are related to female students’ physical activity participation (Wilson et al., 2022). In addition, campus recreational environments may not always match the activity preferences of all students (Wilson et al., 2022; Cao, 2015; Doyle et al., 2019).

Differences in social support may offer another explanation. Prior studies suggest that some female students receive less encouragement for physical activity from peers, families, or schools. This may reduce their confidence or willingness to take part in exercise (Eski̇ler and Küçüki̇bi̇ş, 2019; Sheng et al., 2025). Taken together, these findings suggest that universities may need to pay closer attention to the quality of participation experiences and the support available to female students. Still, these interpretations remain speculative because these contextual factors were not directly assessed in the present study.

### Effects of sports environment, peer support, and perceived physical literacy on physical fitness

This study showed that more favorable perceptions of the campus sports environment and greater peer support were both positively associated with physical fitness. This pattern suggests that better physical infrastructure and a more supportive exercise culture on campus may be related to better physical fitness outcomes among students (Reid et al., 2015). This interpretation is broadly consistent with a socio-ecological perspective, which emphasizes that environmental resources and interpersonal support may jointly shape opportunities for physical activity and related developmental outcomes (Hu et al., 2021; Su et al., 2024; Zhang et al., 2012).

However, environmental and interpersonal factors are unlikely to be the only correlates of physical fitness. Individual-level factors, such as confidence, knowledge, and self-management capacity, may also be important (Demetriou et al., 2018). In the present study, perceived physical literacy showed the strongest correlation with physical fitness, suggesting that students with greater perceived physical literacy also had higher physical fitness scores. Prior research has similarly linked physical literacy to sustained physical activity participation and favorable fitness-related outcomes (Majić et al., 2024). Accordingly, one plausible interpretation is that students with higher perceived physical literacy may be more prepared to engage in physical activity and to navigate exercise-related settings more confidently.

### Chain mediation effects and socio-ecological interpretation

This study further examined the chain mediation mechanism linking perceived campus sports environment, peer support, perceived physical literacy, and physical fitness. After peer support and perceived physical literacy were included in the model, the direct association between perceived campus sports environment and physical fitness was no longer statistically significant, whereas the indirect pathways remained significant. This pattern should not be interpreted as definitive evidence of complete mediation. Because collinearity diagnostics did not indicate problematic multicollinearity, the attenuated direct association is more appropriately interpreted as suggesting that the relationship between perceived campus sports environment and physical fitness may operate largely through indirect pathways, particularly peer support and perceived physical literacy.

Specifically, a more favorable perception of the sports environment may be associated with greater peer interaction and support. Well-maintained facilities and an encouraging atmosphere may create conditions that support collaboration and peer connection, which may in turn strengthen students’ motivation to participate in physical activity (Wu et al., 2024). Previous studies have suggested that a more supportive campus sport environment may be related to students’ sense of belonging, identification with physical activity, interpersonal communication, and mutual support (Li, 2024; Zhang Y. et al., 2022).

Peer support may also be related to perceived physical literacy through multiple pathways. Positive peer interaction may strengthen interest, motivation, confidence, and self-efficacy in physical activity (Douglas et al., 2024; Li, 2024). Knowledge-sharing and collaborative learning may also help students acquire exercise-related knowledge and skills, thereby supporting some cognitive and confidence-related aspects of perceived physical literacy (Luo et al., 2022).

These findings are broadly consistent with socio-ecological systems theory, which suggests that exercise-related outcomes are shaped by interactions among individual, interpersonal, and environmental factors (Ishii et al., 2010; Jie, 2024; Rhodes et al., 2020). A favorable physical environment alone does not automatically lead to healthier behaviors; instead, it provides the contextual conditions that may support healthier behavioral choices. The extent to which environmental opportunities are translated into actual participation may depend in part on whether students have sufficient willingness, capability, and peer support for exercise (Cerin et al., 2008; Graham et al., 2011; Jie, 2024).

Thus, while the campus sports environment may provide a structural basis for healthy behavior, the combined role of social support and perceived physical literacy may help explain how environmental resources are associated with better physical fitness outcomes. These findings underscore the importance of multi-level, integrative strategies in campus sports management and policy-making to support students’ physical fitness more effectively.

### Practical implications

The findings of this study have important implications for the design of campus sport interventions and related policies. First, interventions may benefit from simultaneously improving the physical environment, enhancing social support, and developing students’ physical literacy, rather than focusing on a single dimension. For instance, while improving sports infrastructure, universities should also foster a supportive campus culture by encouraging students to form exercise groups or sports teams and by designating senior students or athletic leaders as role models.

At the individual level, physical literacy should be integrated into both curricular and extracurricular programs to equip students with scientific knowledge and skills, while fostering awareness and confidence for lifelong participation in physical activity. A number of multi-layered strategies have been proposed in the literature, including stimulating intrinsic motivation, enhancing peer encouragement, promoting instructor support, and increasing access to diverse facilities (Xiao et al., 2020). These initiatives, working across individual, interpersonal, and environmental levels, contribute to a healthy ecosystem that supports student engagement in physical activity. When these components work together, they may create a campus setting that better supports students’ physical activity and may also be linked to better physical fitness.

Since the launch of the Sunshine Sports Movement in 2007, China has placed increasing emphasis on improving student physical fitness through school-based physical education. The present findings suggest that, when interpreting or monitoring such policies, it may be useful to consider not only formal requirements, but also whether they are accompanied by supportive campus environments and positive social experiences. Policymakers could usefully assess whether these initiatives are associated with students’ perceptions of the sport environment, peer support, and perceived physical literacy, as these factors may be relevant to later physical fitness (Graham et al., 2011). From this perspective, policy evaluation may benefit from considering not only resource input but also students’ lived experience of the campus sport context.

The gender differences identified in this study warrant attention from university administrators and policymakers. To address the observed gender differences in perceived facility access and peer-support indicators, gender-sensitive strategies should be considered. Universities could expand the range and accessibility of recreational spaces and offer activity options that are more welcoming and accessible to female students. They could also foster a more inclusive sport culture by challenging gender stereotypes and supporting women’s participation across different forms of physical activity. In addition, universities and student associations could highlight female athletes and student leaders as visible role models. Strengthening opportunities for social support, including peer-based participation and mutual encouragement, may be particularly relevant for female students, given their relatively lower peer-support indicators in the present sample. Prior studies have shown that women often face less external support and lower cultural expectations regarding physical activity (Sheng et al., 2025). Against this background, creating environments in which female students feel more comfortable and supported may be especially valuable. Over time, such efforts could improve female students’ perceptions of the sport environment and peer support and may also be associated with more positive participation experiences which may in turn be relevant to physical fitness.

### Limitations and future directions

This study has several limitations. First, although a two-wave prospective observational design was used, the predictors and mediators were assessed concurrently at T1, while physical fitness was measured at T2. Therefore, the findings should not be interpreted as evidence of causal mediation, and the directionality between peer support and physical literacy cannot be established with certainty. Future studies should adopt multi-wave longitudinal or experimental designs to test temporal ordering more rigorously. Second, while the study focused on a theoretically grounded chain model, other potentially relevant factors, such as socioeconomic status, parental education, and the quality of peer relationships, were not included. In addition, physical literacy was assessed using the PPLI, which primarily captures perceived knowledge, communication, and confidence, rather than the full breadth of behavioral participation and objective physical competence. Likewise, peer support was measured using a brief three-item activity-related scale and may not fully reflect the multidimensional nature of peer support. Third, the key psychosocial variables were all assessed using self-report measures at T1, which may increase the risk of common method bias, although Harman’s single-factor test did not indicate a dominant common factor. More robust procedural and statistical approaches, such as multi-informant data or marker-variable strategies, would strengthen future research. Fourth, the sample was drawn from five universities in Hubei Province using convenience sampling. Accordingly, the findings should be generalized cautiously to other regions, institutional contexts, and cultural settings. In addition, the association involving peer support may be shaped in part by the collectivist social context of Chinese universities, where group norms and interpersonal connectedness may play a particularly salient role in exercise-related behavior. Therefore, caution is needed when generalizing these findings to contexts in which peer influence may operate differently. Finally, the present model examined a unidirectional pathway. Future research could test reciprocal or moderated pathways to better capture the dynamic relationships among campus sport environment, physical fitness, and related psychosocial processes.

## Conclusion

In summary, this study, grounded in socio-ecological theory and informed by perspectives on campus sports policy and physical literacy, examined gender-related patterns and multilevel correlates of college students’ physical fitness. The findings underscore the combined relevance of physical infrastructure, social support, and individual literacy in understanding differences in physical fitness. Specifically, the association between perceived campus sports environment and physical fitness was indirectly linked to peer support and perceived physical literacy. For university practitioners and policymakers, these findings suggest the value of strategies that go beyond infrastructure development alone. Institutions may benefit from fostering a positive campus exercise culture and implementing programs that strengthen students’ perceived physical literacy, while paying particular attention to the lower facility-access and peer-support scores reported by female students. Such multi-level efforts may help create campus conditions that are more supportive of physical activity and, potentially, of better physical fitness among college students. Overall, the results support the importance of campus sports environments that are supportive, inclusive, and responsive to students’ social and literacy-related resources.

## Data Availability

The raw data supporting the conclusions of this article will be made available by the authors, without undue reservation.
